# Urinary Glycol Ether Metabolites in Women and Time to Pregnancy: The PELAGIE Cohort

**DOI:** 10.1289/ehp.1206103

**Published:** 2013-07-09

**Authors:** Ronan Garlantézec, Charline Warembourg, Christine Monfort, Laurence Labat, Juha Pulkkinen, Nathalie Bonvallot, Luc Multigner, Cécile Chevrier, Sylvaine Cordier

**Affiliations:** 1 INSERM (Institut national de la santé et de la recherche médicale), U1085 IRSET (Institut de Recherche en Santé, Environnement et Travail), Rennes, France; 2 University of Rennes I, Rennes, France; 3 EHESP (École des hautes études en santé publique) School of Public Health, Rennes, France; 4 Toxicology and Genopathy Laboratory, CHRU (Centre Hospitalier Régional Universitaire) Lille, Lille, France; 5 School of Pharmacy, University of Eastern Finland, Kuopio, Finland

## Abstract

Background: Glycol ethers are present in a wide range of occupational and domestic products. Animal studies have suggested that some of them may affect ovarian function.

Objective: We examined the relation between women’s exposure to glycol ethers and time to pregnancy.

Methods: We used chromatography coupled to mass spectrometry to measure eight glycol ether metabolites in urine samples from randomly selected women in the PELAGIE mother–child cohort who had samples collected before 19 weeks of gestation. Using time to pregnancy information collected at the beginning of the pregnancy (women were asked how many months it took for them to conceive), we estimated associations between metabolite levels and time to pregnancy in 519 women with complete data using discrete-time Cox proportional hazards models to adjust for potential confounders.

Results: We detected glycol ether metabolites in 6% (for ethoxyacetic acid) to 93% (for phenoxyacetic and butoxyacetic acids) of urine samples. Phenoxyacetic acid was the only metabolite with a statistically significant association with longer time to pregnancy [fecundability OR = 0.82; 95% CI: 0.63, 1.06 for the second and third quartile combined; fecundability OR = 0.70; 95% CI: 0.52, 0.95 for a fourth-quartile (≥ 1.38 mg/L) vs. first-quartile concentration (< 0.14 mg/L)]. This association remained stable after multiple sensitivity analyses.

Conclusion: Phenoxyacetic acid, which was present in most of the urine samples tested in our study, was associated with increased time to pregnancy. This metabolite and its main parent compound, 2-phenoxyethanol, are plausible causes of decreased fecundability, but they may also be surrogates for potential coexposures to compounds frequently present in cosmetics.

Citation: Garlantézec R, Warembourg C, Monfort C, Labat L, Pulkkinen J, Bonvallot N, Multigner L, Chevrier C, Cordier S. 2013. Urinary glycol ether metabolites in women and time to pregnancy: the PELAGIE cohort. Environ Health Perspect 121:1167–1173; http://dx.doi.org/10.1289/ehp.1206103

## Introduction

Organic solvents are present in a wide variety of occupational and domestic products, including cleaning agents, cosmetics (i.e., makeup, creams, soaps, and deodorants), paints, and varnishes. Several previous studies have reported associations between women’s occupational exposure to solvents and fertility impairment, although the results were not all concordant (Mendola et al. 2008). Limitations of such studies have included the lack of objective assessment of exposure to specific solvents and a failure to study nonoccupational exposure. Biological monitoring of chemicals is a useful alternative because it provides an integrative and objective exposure measurement (Angerer et al. 2007; Calafat et al. 2006).

From the solvents suspected of impairing female fertility, we decided to focus on glycol ethers (GEs), a family of oxygenated solvents that includes > 30 different ethers of ethylene glycol and propylene glycol. GEs represented 5% of the total volume of solvents used in France in 2004 (Triollet 2005). Chemical analytical methods were available to measure eight metabolites of the GEs most frequently used in France at the time of the study [Agence française de sécurité sanitaire de l’environnement et du travail (AFSSET) 2008] (Table 1). Animal studies have reported that several GEs may affect ovarian function: 2-methoxyethanol (EGME), 2-ethoxyethanol (EGEE), 2-butoxyethanol (EGBE), 2-phenoxyethanol (EGPhE), and 1,2-bis(methoxyethoxy)ethane (TEGDME) (reviewed by Multigner et al. 2005). In occupational epidemiologic studies in women conducted in the semiconductor industry, where EGME and EGEE were the main GEs used, GE exposure was associated with prolonged time to pregnancy (TTP) (Chen et al. 2002; Correa et al. 1996; Eskenazi et al. 1995; Gray et al. 1996) and with prolonged menstrual cycles (Hsieh et al. 2005). However, use of EGME and EGEE has been restricted in France since 1999 (AFSSET 2008). Neither EGBE nor EGPhE—both frequently present in consumer products and suspected of impairing women’s fertility—has ever been studied in women.

**Table 1:** Primary products in France that contain GEs (2000–2006) and their main urinary metabolites.

Products	GEs^*a*^	Metabolites
Cosmetics	EGPhE	PhAA
	EGBE	BAA
	DEGBE	BEAA, BAA
	DEGEE	EEAA, EAA
Drugs	EGPhE	EEAA, EAA
	EGPhE	PhAA
Cleaning agents	EGPhE	EEAA, EAA
	DEGBE	BEAA, BAA
	EGBE	BAA
	PGME	2-MPA^*b*^
	DEGME	MEAA, MAA
	TEGME	MEAA, MAA
	TEGDME	MAA
Biocides	EGPhE	2-MPA^*b*^
	EGBE	BAA
	DEGBE	BEAA, BAA
	DEGEE	EEAA, EAA
	EGPhE	PhAA
Paints, varnishes, and inks	EGPhE	2-MPA^*b*^
	EGBE	BAA
	DEGBE	BEAA, BAA
	DEGEE	EEAA, EAA
	TEGEE	EEAA, EAA
	EGnPE	PAA
Abbreviations: BAA, 2-butoxyacetic acid; DEGBE, 2-(2-butoxy­ethoxy)ethanol; DEGEE, 2-(2-ethoxy­ethoxy)ethanol; DEGME, 2-(2-methoxyethoxy)ethanol; EAA, ethoxy­acetic acid; EEAA, ethoxyethoxy­acetic acid; EGBE, 2-butoxy­ethanol; EGEE, 2-ethoxy­ethanol; EGME, 2-methoxy­ethanol; EGnPE, 2-propoxy­ethanol; EGPhE, 2-phenoxy­ethanol; MAA, methoxy­acetic acid; MEAA, methoxy­ethoxy­acetic acid; 2-MPA, 2-methoxy­proprionic acid; PAA, *n*-propoxyacetic acid; PGME, methoxy­propanol; PhAA, phenoxy­acetic acid; TEGDME, 1,2-bis(methoxyethoxy)ethane; TEGEE, 2-(2-(2-ethoxy­ethoxy)ethoxy)ethanol; TEGME, 2-(2-(2-methoxy­ethoxy)ethoxy)ethanol. Data from AFSSET (2008).^***a***^GEs are presented for products by most to least frequently used. ^***b***^Metabolite derived from minor β isomer of PGME.		

The objective of our work was to study the relation between both nonoccupational and occupational exposure to GEs in women and TTP.

## Materials and Methods

*Study population.* The PELAGIE (Perturbateurs endocriniens: Étude Longitudinale sur les Anomalies de la Grossesse, l’Infertilité et l’Enfance) cohort study, described elsewhere (Garlantézec et al. 2009), included 3,421 pregnant women in three districts of Brittany (northwestern France) between 2002 and 2006. Women were recruited by their gynecologists, obstetricians, or ultrasonographers at visits for prenatal care before 19 weeks of gestation and were followed through the end of pregnancy. Participants provided informed consent for participation, and the INSERM (Institut national de la santé et de la recherche médicale) ethics committee approved the study procedures.

At inclusion, women completed a self-administered questionnaire that covered family social and demographic characteristics, diet, and lifestyle. Each woman returned the questionnaire by mail to our laboratory along with a urine sample (first morning void) in a 10-mL test tube (95 × 16-mm polypropylene, with wing plug). Upon arrival at the laboratory, urine samples were frozen at –20°C until analysis.

Because of costs, the biomonitoring study included only a randomly selected sample (*n* = 609) of the women in the PELAGIE study.

**Figure 1:**
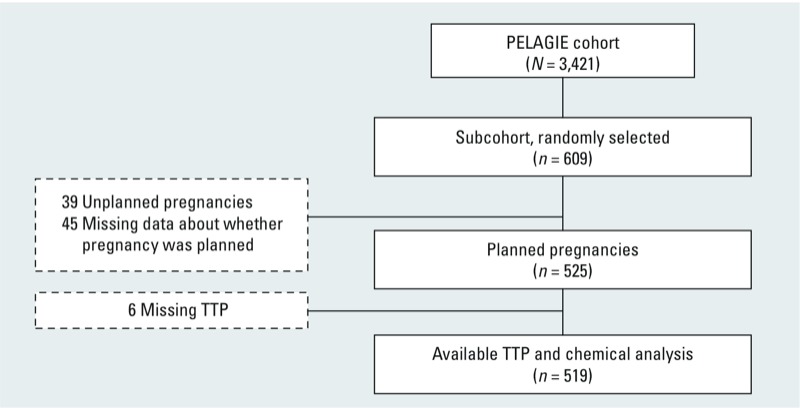
Flow diagram showing study sample selection.

*Assessment of TTP.* TTP was based on the answer to the question “How long did it take you to become pregnant?” (in months) from the self-administered questionnaire collected at inclusion. The questionnaire also asked about contraceptive use, voluntary interruption of contraceptive use (to assess whether the pregnancy was planned), fertility medication (to censor at time of conception), and reproductive history (to validate the self-reported TTP by verifying the absence of any interim miscarriage during the TTP). Among women with urine analyses, TTP was available for 519 (85%) (Figure 1). TTP was unavailable when the pregnancy was unplanned (*n* = 39), if it was not clear whether the pregnancy was planned or not (*n* = 45), or if TTP data were missing (*n* = 6).

*Chemical analysis of GE metabolites.* Chemical analyses were performed at the Toxicology and Genopathy Laboratory at CHRU (Centre Hospitalier Régional Universitaire) Lille following the method of Labat et al. (2008). GE metabolites were measured by gas chromatography coupled to mass spectrometry, and detection was performed in negative ionization mode with methane in full-scan acquisition between 85 and 152 *m/z*. This method allowed for the simultaneous analysis of eight alkoxycarboxylic acids, the main metabolites of the GEs in use in Europe at the time of urine collection (Table 1): methoxyacetic acid (MAA), methoxyethoxyacetic acid (MEAA), ethoxyacetic acid (EAA), ethoxyethoxyacetic acid (EEAA), 2-butoxyacetic acid (BAA), *n*-propoxyacetic acid (PAA), phenoxyacetic acid (PhAA), and 2-methoxypropionic acid (2-MPA). This method yields coefficients of variation < 10% at 0.10 mg/L (Labat et al. 2008). The method is linear (*r*^2^ = 0.99) from 0.05 to 2.0 mg/L for all of the alkoxycarboxylic acids, with a limit of detection (LOD) of 0.05 mg/L.

The influence of sampling conditions, including transportation time at room temperature, duration of storage at –20°C, and type of acidic stabilizer [hydrochloric acid (HCl) or nitric acid (HNO_3_)] on urinary measurements, and of individual parameters (urinary creatinine level and gestational age at inclusion) was reported previously (Cordier et al. 2012). In that study, the BAA detection rate decreased with the number of days at ambient temperature when HCl was used as a stabilizer, but not when HNO_3_ was used; therefore, here we present results for BAA only for the subset of 412 women whose samples were stabilized with HNO_3_. Statistical analysis performed by Cordier et al. (2012) showed that creatinine level was associated with MAA, MEAA, EEAA, BAA, and PhAA, and that transportation time appeared to influence the levels of BAA and the detection of 2-MPA. Duration of storage [median: 1,090 days; quartile (Q) 1, 858 days; Q3, 1,377 days] was associated with levels of MEAA, EEAA, PAA, and 2-MPA (Cordier et al. 2012). Thus, we considered these covariates (creatinine level, gestational age at inclusion, transport duration, and duration of storage between collection and analysis) to be potential confounders.

*Statistical analysis.* Each metabolite with a detection rate < 50% was categorized in two classes (< LOD and ≥ LOD). Other metabolites were categorized in three classes (< 25th percentile, 25th–74th percentile, ≥ 75th percentile) and were also modeled as ordinal variables coded using integer values (0, 1, 2) to perform trend tests. Each metabolite with a detection rate > 30% was also treated as continuous with a distribution-based multiple imputation method for handling values < LOD (Jin et al. 2011; Lubin et al. 2004). Assuming a lognormal distribution of each metabolite, this method uses maximum likelihood estimates to estimate distribution parameters (Jin et al. 2011; Lubin et al. 2004). We used the Cox proportional hazards models adapted for discrete-time data to estimate fecundability odds ratios (fORs) associated with urinary biomarkers of exposure to GE metabolites. The fOR estimates the odds of achieving pregnancy in any given month, conditional on not having become pregnant in a previous cycle. An fOR of < 1 indicates longer TTP and decreased fecundability. When TTP was > 12 months (13% of women, *n* = 66), the value was censored at 12 months. When couples who sought medical assistance due to difficulty in becoming pregnant reported TTP < 12 months (3% of women, *n* = 16), we censored at their self-reported TTP, considering it as a minimal one (as if they had not used medical assistance).

Covariates systematically included in the models were well-established infertility risk factors that were self-reported in our study: maternal age (years, continuous), body mass index (BMI; < 18.5 kg/m^2^, 18.5–25 kg/​m^2^, ≥ 25 kg/m^2^), maternal smoking status when first attempting pregnancy (yes/no), and oral contraceptive use before attempting pregnancy (yes/no). We observed that the probability in our cohort of conceiving in the first 2–3 months was higher for women who had not used oral contraception immediately before attempting pregnancy than for women who did. After 3 months, these two groups no longer differed substantially. This finding, which has also been reported by others (Axmon et al. 2006), led us to stratify our analyses for oral contraceptive use with a stratified Cox model.

We also evaluated other potential covariates: year of inclusion (2002, 2003, 2004, 2005–2006), district of residence (Ille-et-Vilaine, Côtes d’Armor, others), rural/urban residence area, season as a time-varying covariate, self-reported maternal education level (primary/secondary, baccalaureate, postbaccalaureate), coffee consumption at inclusion (no more than once a day, at least twice a day), fish consumption (less than monthly, 1–4 times a month, at least twice a week), shellfish consumption (less than twice a month, 2–8 times a month, at least twice a week), paternal occupational exposure to solvents at inclusion according to a job-exposure matrix (Ferrario et al. 1988) (unemployed, none, medium, high), and variables describing urine sampling conditions. These variables were to be included in the models if they modified the fOR by ≥ 10%, but none met this criterion. In addition, when we observed correlations between metabolites and between TTP and at least one of these metabolites, we ran models that simultaneously included these metabolites.

Correlations between the urinary metabolites were analyzed with chi-square or Fisher’s exact tests, Tobit regression, or Spearman rank correlations, depending on the level of detection. Statistical significance was defined as *p* < 0.05.

*Sensitivity analyses.* The first set of sensitivity analyses examined the consistency of our findings in light of potential validity issues for TTP studies. As recommended by previous studies (Joffe et al. 2005; Weinberg et al. 1994), in addition to the main analyses, we report estimates from analyses *a*) restricted to the 237 primiparous women, *b*) excluding 113 women who reported conceiving in the first month, *c*) including 39 pregnancies resulting from contraceptive failure (TTP was assigned a value of 0), *d*) excluding 37 women seeking fertility treatment, and *e*) changing the censoring time from 12 months to 7, 10, or 15 months.

## Results

The 519 eligible women in our study had a mean age of 29.4 years when they began attempting to conceive, and their educational attainment was high: Around 63% of participants had received some postbaccalaureate education (Table 2). Mean gestational age at inclusion was 9.6 weeks (Q1, 7.7 weeks; Q3, 13.3 weeks). More than half of the participants already had a child, and around 16% had previously miscarried. Most (75%) of the women had used oral contraceptives before trying to conceive, and around 7% had undergone fertility treatment.

**Table 2:** Median time to pregnancy and crude fOR (fecundability odds ratio) according to population characteristic.

Characteristic	*n* (%)	Median TTP(Q1–Q3)^*a*^	CrudefOR (95% CI)^*a*^	*p*-Value
Total	519 (100.0)	3 (2–7)
Year of inclusion	0.48
2002	62 (12.0)	3 (1–6)	Reference
2003	205 (39.5)	3 (2–7)	0.77 (0.55, 1.09)
2004	188 (36.2)	3 (1–6)	0.88 (0.62, 1.25)
2005–2006	64 (12.3)	3 (2–6)	0.88 (0.57, 1.33)
District of residence	0.84
Ille-et-Vilaine	348 (67.1)	3 (2–6.5)	Reference
Côtes d’Armor	130 (25.0)	3 (2–6)	1.00 (0.78, 1.27)
Others	41 (7.9)	4 (2–7)	0.89 (0.60, 1.32)
Educational level	0.06
Primary/secondary	89 (17.2)	5 (2–6.5)	Reference
Baccalaureate	103 (19.9)	3 (2–10)	0.95 (0.67, 1.34)
Postbaccalaureate	326 (62.9)	3 (2–6)	1.26 (0.94, 1.68)
Missing	1	9	—
Maternal age (year) at pregnancy attempt	0.21
< 25	61 (11.7)	3 (2–8)	Reference
25–30	206 (39.7)	3 (2–7)	1.07 (0.75, 1.52)
30–35	195 (37.6)	3 (2–6)	1.34 (0.94, 1.92)
≥ 35	57 (11.0)	3 (2–7)	1.15 (0.74, 1.79)
Mean ± SD	29.4 ± 4.0
Prepregnancy BMI (kg/m²)	0.29
< 18.5	39 (7.6)	3 (1–5)	1.14 (0.75, 1.73)
18.5–25	399 (77.3)	3 (2–6)	Reference
≥ 25	78 (15.1)	3 (2–8)	0.81 (0.60, 1.09)
Missing	3	6 (1–12)	—
Mean ± SD	22.1 ± 3.4
Gestational age at inclusion (weeks)	0.25
< 10	219 (42.2)	3 (2–7)	Reference
10–13	240 (46.2)	4 (2–7)	0.94 (0.75, 1.18)
> 13	60 (11.6)	3 (2–5)	1.27 (0.89, 1.81)
Mean ± SD	9.6 ± 2.5
Parity	0.02
0	237 (45.7)	3.5 (2–8)	Reference
1	193 (37.2)	3 (2–6)	1.26 (1.00, 1.59)
≥ 2	89 (17.2)	3 (1–6)	1.48 (1.09, 1.99)
History of miscarriage	0.004
No	434 (83.6)	3 (2–6)	Reference
Yes	85 (16.4)	4 (2 –10)	0.65 (0.49, 0.87)
Oral contraceptive use before pregnancy attempt	0.58
No	122 (23.5)	3 (1–7)	Reference
Yes	396 (76.5)	3 (2–6)	1.07 (0.84, 1.38)
Missing	1	1 (1–1)	—
Maternal smoking at pregnancy attempt	0.22
No	362 (70.8)	3 (2–6)	Reference
Yes	149 (29.2)	3 (2–8)	0.86 (0.69, 1.09)
Missing	8	6 (4–8.5)	—
Coffee consumption at inclusion	0.94
≤ Once per day	366	3 (2–7)	Reference
At least twice per day	116	3 (2–6.5)	0.99 (0.78, 1.27)
Fish consumption	0.81
Less than monthly	102 (19.7)	3 (2–7)	Reference
1–4 times per month	263 (50.9)	3 (2–7)	1.04 (0.79, 1.37)
At least twice per week	152 (29.4)	3 (2–6)	1.10 (0.81, 1.50)
Missing	2	3 (3–3)	—
Shellfish consumption	0.36
Less than twice per month	353 (68.0)	3 (2–6)	Reference
2–8 times per month	126 (24.3)	3 (2–7)	0.98 (0.76, 1.26)
At least twice per week	40 (7.7)	4 (2–8)	0.74 (0.50, 1.12)
Marital status	0.30
Live alone	5 (1.0)	3 (2–6)	0.57 (0.20, 1.64)
Lives with partner	513 (99.0)	5 (4–12)	Reference
Missing	1	17	—
Fertility treatment
No	482 (92.9)		—
Yes	37 (7.1)		—
CI, confidence interval. ^***a***^Based on data from women who did not seek medical assistance to achieve pregnancy (*n *= 482).				

As we expected, the entire cohort and the random subcohort were similar for these characteristics (see Supplemental Material, Table S1). The median TTP was 3 months (Q1–Q3, 2–7 months) (Table 2). Increased parity and unemployment were statistically associated with a reduced TTP (fOR > 1.0), and a history of miscarriage was significantly associated with a longer TTP (fOR < 1.0).

As shown in Table 3, BAA and PhAA were detected in 93% of the women’s urinary samples and MEAA in more than half. The highest median concentration (estimated from all detected values) was observed for PhAA (0.48 mg/L).

**Table 3:** Distribution of GE metabolites (alkoxycarboxylic acids) in urine samples from 519 women.

Alkoxycarboxylic acid (LOD = 0.05 mg/L)	Values ≥ LOD^*a*^[*n* (%)]	Percentile (mg/L)			Maximum (mg/L)	Median (values ≥ LOD) (mg/L)
		25th	50th	75th		
MAA	146 (28)	< LOD	< LOD	0.06	2.97	0.09
MEAA	289 (56)	< LOD	0.06	0.26	3.90	0.23
EAA	21 (4)	< LOD	< LOD	< LOD	0.62	0.06
EEAA	92 (19)	< LOD	< LOD	< LOD	30.00	0.09
PAA	61 (12)	< LOD	< LOD	< LOD	0.24	0.07
BAA^*a*^	385 (93)	0.085	0.12	0.16	0.62	0.13
PhAA	484 (93)	0.14	0.38	1.38	36.00	0.48
2-MPA	29 (6)	< LOD	< LOD	< LOD	0.76	0.13
Abbreviations: BAA, butoxy­acetic acid; EAA, ethoxyacetic acid; EEAA, ethoxyethoxyacetic acid; MAA, methoxyacetic acid; MEAA, methoxy­ethoxy­acetic acid; 2-MPA, 2-methoxyproprionic acid; PAA, *n*-propoxy­acetic acid; PhAA, phenoxy­acetic acid. ^***a***^Excluding 107 samples that were stabilized with HCl.						

When we compared the likelihood of detection (i.e., concentration ≥ LOD versus < LOD) for metabolites that were detected in < 30% of samples (specifically, MAA, EAA, EEAA, PAA, and 2-MPA), only PAA detection and 2-MPA detection were significantly correlated, although we did observe nonsignificant associations between MAA and MPA (*p* = 0.08) and between EAA and EEAA (*p* = 0.09) (see Supplemental Material, Table S2). When we evaluated correlations between detection of the metabolites above and the concentration of GE metabolites detected in the majority of samples (MEAA, BAA, and PhAA), we found significant correlations between MAA ≥ LOD and MEAA concentration, EEAA ≥ LOD and PhAA concentration, MPA ≥ LOD and the concentrations of MEAA and BAA, and PAA ≥ LOD and BAA concentration (see Supplemental Material, Table S3). However, there were no correlations between MEAA, BAA, and PhAA concentrations (see Supplemental Material, Table S4).

Table 4 shows the associations between TTP and each GE urinary metabolite. Fecundability increased with PAA detection [fOR = 1.30; 95% confidence interval (CI): 0.94, 1.80]. PhAA was the only metabolite significantly associated with TTP: a Q4 concentration of PhAA (≥ 1.38 mg/L) was associated with a 30% decrease (fOR = 0.70; 95% CI: 0.52, 0.95) in the odds of becoming pregnant each month, compared with the Q1 concentration (< 0.14 mg/L). Moreover, we observed a statistically significant dose–response trend in which fecundability decreased as PhAA levels increased (*p*-trend = 0.02). This result was confirmed by the analysis with PhAA levels treated as a continuous variable (fOR for a 1-mg/L increase in PhAA = 0.95; 95% CI: 0.90, 1.00). Because of the positive correlation between PhAA levels and EEAA detection, we further adjusted for EEAA, but the association with PhAA remained unchanged (fOR for a 1-mg/L increase in PhAA = 0.95; 95% CI: 0.90, 1.00).

**Table 4:** Relation between GE metabolites (alkoxycarboxylic acids) in urine samples from 519 women and TTP.

Metabolite level	Crude			Adjusted^*a*^		
	*n*	fOR (95% CI)	*p*-Trend	*n*	fOR (95% CI)	*p*-Trend
MAA
< LOD	373	Reference		365	Reference
≥ LOD	146	1.05 (0.84, 1.33)		142	1.10 (0.87, 1.39)
MEAA			0.31			0.29
< LOD	230	Reference		224	Reference
LOD to < 0.23 mg/L	144	1.22 (0.95, 1.56)		140	1.24 (0.96, 1.61)
≥ 0.23 mg/L	145	1.12 (0.87, 1.43)		143	1.12 (0.87, 1.45)
Continuous^*b*^	519	1.02 (0.78, 1.32)		507	1.03 (0.77, 1.37)
EAA
< LOD	498	Reference		486	Reference
≥ LOD	21	0.90 (0.52, 1.56)		21	0.94 (0.54, 1.63)
EEAA
< LOD	427	Reference		416	Reference
≥ LOD	92	0.88 (0.67, 1.16)		91	0.88 (0.66, 1.17)
PAA
< LOD	458	Reference		447	Reference
≥ LOD	61	1.25 (0.91, 1.72)		60	1.30 (0.94, 1.80)
BAA			0.96			0.86
Q1 (< 0.09)	103	Reference		101	Reference
Q2–Q3 (0.09 to < 0.16 mg/L)	192	0.98 (0.74, 1.31)		189	0.92 (0.68, 1.24)
Q4 (≥ 0.16 mg/L)	117	1.01 (0.73, 1.39)		112	0.96 (0.70, 1.34)
Continuous^*b*^	412	1.95 (0.39, 9.79)		402	1.44 (0.28, 7.55)
PhAA			0.02			0.02
Q1(< 0.14 mg/L)	128	Reference		126	Reference
Q2–Q3 (0.14 to < 1.38 mg/L)	261	0.81 (0.63, 1.05)		255	0.82 (0.63, 1.06)
Q4 (≥ 1.38 mg/L)	130	0.70 (0.52, 0.94)		126	0.70 (0.52, 0.95)
Continuous^*b*^	519	0.95 (0.90, 1.00)^*c*^		507	0.95 (0.90, 1.00)^*c*^
2-MPA
< LOD	490	Reference		478	Reference
≥ LOD	29	1.05 (0.66, 1.65)		29	1.10 (0.69, 1.75)
Abbreviations: BAA, butoxy­acetic acid; EAA, ethoxyacetic acid; EEAA, ethoxyethoxyacetic acid; MAA, methoxyacetic acid; MEAA, methoxy­ethoxy­acetic acid; 2-MPA, 2-methoxyproprionic acid; PAA, *n*-propoxy­acetic acid; PhAA, phenoxy­acetic acid. LOD = 0.05 mg/L. ^***a***^Adjusted for maternal age at pregnancy attempt (years), prepregnancy BMI (< 18.5 kg/m², 18.5–25 kg/m², ≥ 25 kg/m²), maternal smoking at pregnancy attempt (no/yes), and oral contraceptive use before pregnancy attempt (no/yes). ^***b***^Estimated by multiple imputation for non­detects. ^***c***^For 95% CIs, values of 1.00 were excluded before rounding to two decimal places.						

**Figure 2:**
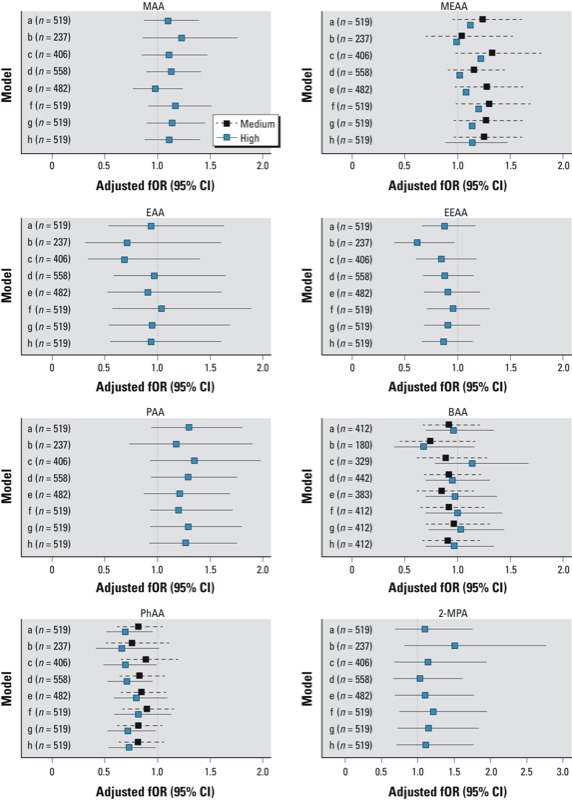
Adjusted fORs and 95% CIs for association of medium and high levels of alkoxycarboxylic acids with TTP compared with the lowest levels, according to various sensitivity analyses: *a*) main population; *b*) limited to 237 primiparous women; *c*) excluding 113 women who conceived in the first month of trying; *d*) including 39 pregnancies resulting from contraceptive failure (TTP = 0); *e*) excluding 37 women who used fertility treatment; *f*) censored at 7 months; *g*) censored at 10 months; and *h*) censored at 15 months. For metabolites analyzed in three classes (MEAA, BAA, PhAA), medium and high levels are shown in Table 4; for metabolites analyzed in two classes (MAA, EAA, EEAA, PAA, and 2‑MPA), high levels are ≥ LOD. Abbreviations: BAA, butoxy­acetic acid; EAA, ethoxy­acetic acid; EEAA, ethoxy­ethoxy­acetic acid; MAA, methoxy­acetic acid; MEAA, methoxy­ethoxy­acetic acid; 2‑MPA, 2‑methoxy­proprionic acid; PAA, n-propoxy­acetic acid; PhAA, phenoxy­acetic acid.

The sensitivity analyses of subsets of women and the influence of different censoring times for TTP (Figure 2) yielded similar conclusions for most of the metabolites. The association of longer TTP with a higher PhAA level remained stable. EEAA detection was significantly associated with a longer TTP among primiparous women.

## Discussion

In our study population, concentrations of PhAA (the primary metabolite of EGPhE) in urine samples collected at 9.6 weeks of gestation (on average) were associated with longer TTP. This association remained stable after sensitivity analyses. The other GE urinary metabolites measured in our study were not associated with reduced fecundability.

This association of PhAA concentration with TTP may be a chance finding due to multiple comparisons, or it could be a result of uncontrolled confounding or other sources of bias. However, it has some biological plausibility. The high detection rate of PhAA (93%) in our population is consistent with the frequent use of EGPhE, its main precursor, in cosmetics. EGPhE is also present in pharmaceutical products (AFSSET 2008; INSERM 2006) and biocides (AFSSET 2008), and PhAA is used as a food flavoring agent [Joint Food and Agriculture Organization of the United Nations/World Health Organization Expert Committee on Food Additives (JECFA) 2002]. However, these three sources are probably negligible compared with its cosmetic uses [Agence nationale de sécurité du médicament et des produits de santé (ANSM) 2012]. According to French surveys (AFSSET 2008; INSERM 2006), at the time of our study EGPhE was present in at least 50% of perfumes, creams, lotions, makeup, and hair products (except dyes), for example, but it was present in only 15 specific individual pharmaceutical products (of all drugs authorized for sale in France) and in < 1% of biocides (AFSSET 2008; INSERM 2006). Moreover, a study by the French drug and cosmetic safety agency (ANSM 2012) estimated that EGPhE intake in cosmetics reached 2 mg/kg/day with daily use of nonrinsed cosmetics (i.e., creams, lotions, powders, perfumes, lipsticks, nail polish, and eye and facial makeup) compared with 0.3 mg/kg/day among drug users and 0.004 mg/kg/day from food (AFSSET 2008; JECFA 2002). EGPhE has been reported in some cleaning products in the United States (Scognamiglio et al. 2012), but this does not seem to be the case in France (AFSSET 2008).

An expert review from INSERM (Multigner et al. 2005) stated that EGPhE was one of five GEs (EGME, EGEE, EGBE, and TEGDME) likely to impair ovarian function (INSERM 1999, 2006). The evaluation of EGPhE was based on a single study in mice, which reported a decreased number of live pups in the exposed group (Heindel et al. 1990). Our results are consistent with the INSERM evaluation for EGPhE, but they do not suggest that the other tested GEs (EGME, EGEE, EGBE, and TEGDME) have an impact on female fecundability at the levels observed in our study. We have no explanation for the nonsignificant decrease in TTP in association with PAA ≥ LOD in urine samples collected during pregnancy other than chance or uncontrolled bias. To our knowledge, this GE has never been studied in relation to fertility in animal studies.

The only epidemiological studies that have previously evaluated the association between GE exposure and female fertility were performed in the semiconductor industry (Chen et al. 2002; Correa et al. 1996; Eskenazi et al. 1995; Gray et al. 1996), where the principal GEs used were EGME, EGEE, and 1,2-dimethoxyethane. In that occupational setting, prolonged TTP was associated with exposure to these GEs, which was estimated using indirect methods (job title, tasks, and expert assessment). However estimates from those studies may be biased due to exposure misclassification or frequent coexposure to other potential reprotoxic chemicals (i.e., arsenic, isopropyl alcohol, hydrofluoric acid, phosphorous compounds, and xylene) in this occupational setting (Cordier and Multigner 2005). In the present study, MAA and EAA (the main metabolites of the three GEs commonly used in the semiconductor industry) were not frequently detected and were not associated with TTP. However, the regulation of these GEs in Europe since 1999 and the absence from our study of any women working in the semiconductor industry, where exposure to these GEs is high (mean at the end of the workday in France: MAA, 39.2 mg/L; EAA, 14.2 mg/L) (AFSSET 2008), may explain the low presence of their metabolites in our study population and the lack of associations with TTP.

Measurement of urinary alkoxycarboxylic acids is the method of choice for monitoring occupational exposure to GEs (Laitinen and Pulkkinen 2005; Lauwerys et al. 2007); because this method has the capacity to measure exposure from all sources, it could be used to monitor the general population. The metabolites measured in our study have half-lives ranging from 6 to 80 hr and therefore represent recent exposures only. However, for products used daily in the workplace or at home (such as cosmetics), we believe they may reasonably be assumed to represent regular individual exposure before pregnancy, despite the fact that urine collection took place in early pregnancy. Changes in work environment at the beginning of pregnancy are not likely because they are not mandatory for occupations such as health care workers and beauticians or hairdressers, occupations likely to explain most of the occupational GE exposure in our cohort (Cordier et al. 2012). However we cannot exclude possible changes in behavior during pregnancy, in particular, changes in the use of cosmetics (i.e, lotions used to reduce the occurrence of stretch marks although these problems are likely to occur later in pregnancy). Despite the widespread use and potential toxicity of GEs, no one has, to our knowledge, studied their toxicokinetic variability, including during pregnancy. Accordingly, it is possible that our biomonitoring, based only a single urine sample, did not capture total intraindividual variability. Urine PhAA concentrations were significantly associated with only one occupational group in our study population, hairdressers and beauticians (Cordier et al. 2012). Although we did not collect data on personal cosmetic use, we believe it is likely to explain most of the exposure to EGPhE, because only 12 participants reported working as hairdressers and cosmeticians and the association between PhAA and TTP persisted after excluding them from the analysis (fOR = 0.72; 95% CI: 0.53, 0.98 for the Q4 concentration of PhAA compared with Q1).

Although we believe that an effect of EGPhE or its metabolite PhAA on TTP is biologically plausible, we cannot rule out the possibility that urine PhAA concentrations were acting as a proxy marker of exposure to other chemicals, including preservatives frequently present in cosmetics, such as phthalates or parabens. Among phthalates that have been present in cosmetics, di-*n*-butyl phthalate (DBP) has been associated with a decrease in fertility in the female rat (Gray et al. 2006), but this phthalate was banned in cosmetics in France in 2004. A French survey (ANSM 2012) showed that EGPhE and parabens are frequently present together in cosmetics. Parabens were not measured in the urinary samples in our study. Nevertheless, although some parabens (methyl, butyl, benzyl, and isobutylparaben) have been associated with uterotrophic effects in mice or rats at high doses, there is no current evidence that parabens might impair female fertility (Boberg et al. 2010).

Statistical power to detect an effect on fecundability was reduced by our study design: Because our population was limited to pregnant women, it underrepresented less fertile couples and possibly led to the underestimation of effects. The short recall time (during the first 4 months of pregnancy) compensated in part for the retrospective collection of TTP data (Zielhuis et al. 1992). The consistent results of the multiple sensitivity analyses we performed, in accordance with recommendations for addressing other possible biases related to this study design (Joffe et al. 2005; Weinberg et al. 1994), reduce but do not eliminate concerns about the potential influences of these biases on our results.

Because parity and miscarriage might reflect a certain degree of fertility, we chose in advance not to adjust for them. If an association existed, they might be seen as exposure consequences and then be considered as intermediate factors in the study of the relation between exposure and subfertility.

## Conclusion

Among all GEs measured in urine samples collected during pregnancy, we found evidence of longer TTP in association with PhAA. PhAA, which was widely detected in our study population, and its main precursor (EGPhE) are biologically plausible causes of decreased fecundability, but this biomarker may also be a surrogate for co-exposures frequently present in cosmetics. Finally, given the inherent limitations of retrospective TTP studies, further prospective studies on this topic are warranted.

## Supplemental Material

(635 KB) PDF
