# Supplementary material for: Urinary Glycol Ether Metabolites in Women and Time to Pregnancy: The PELAGIE Cohort
Source: Environ Health Perspect. 2013 Jul 9;121(10):1167–73. doi: 10.1289/ehp.1206103 (PMC3801453; doi:10.1289/ehp.1206103)
Supplement: (635 KB) PDF [file ehp.1206103.s001.pdf]

Supplemental Material

Urinary Glycol Ether Metabolites in Women and Time to Pregnancy: The PELAGIE Cohort

Ronan Garlantézec, Charline Warembourg, Christine Monfort, Laurence Labat, Juha Pulkkinen, Nathalie Bonvallot, Luc Multigner, Cécile Chevrier, Sylvaine Cordier

Table of Contents

- Supplemental Material, Table S1: Comparison of the entire cohort and the subcohort used for GE measurements according to maternal characteristics considered in the time-to-pregnancy analyses. p.2
- Supplemental Material, Table S2: relation between detection of metabolites detected in less than half of the sample. p.4
- Supplemental Material, Table S3: Relation between ≥ LOD for metabolites detected in less than half the sample and concentration for metabolites detected in more than half the sample. p.5
- Supplemental Material, Table S4: Correlations between urine concentration of MEAA, BAA and PhAA. p.6

Supplemental Material, Table S1. Comparison of the entire cohort and the subcohort used for GE measurements according to maternal characteristics considered in the time-to-pregnancy analyses

	Cohort with TTP not included in the biomonitoring (n=2437) n (%)	Subcohort with TTP (n=519) n (%)	p value
**Year of inclusion**			0.38
2002	292 (12.0)	62 (12.0)	
2003	904 (37.1)	205 (39.5)	
2004	871 (35.7)	188 (36.2)	
2005-2006	370 (15.2)	64 (12.3)	
**District of inclusion**			0.26
Ille-et-Vilaine	1547 (63.5)	348 (67.1)	
Finistère	658 (27.0)	130 (25.0)	
Côtes d’Armor	232 (9.5)	41 (7.9)	
**Education level**			0.52
Primary/Secondary	407 (16.7)	89 (17.2)	
Baccalaureate	437 (18.0)	103 (19.9)	
Post-secondary	1591 (65.3)	326 (62.9)	
Missing	2	1	
**Maternal age at pregnancy attempt (years)**			0.55
<25	269 (11.0)	61 (11.7)	
25-30	1040 (42.7)	206 (39.7)	
30-35	849 (34.8)	195 (37.6)	
≥35	279 (11.5)	57 (11.0)	
**Gestational age at inclusion (weeks)**			0.17
<10	925 (38.0)	219 (42.2)	
10 – 13	1232 (50.5)	240 (46.2)	
> 13	280 (11.5)	60 (11.6)	
**Pre-pregnancy BMI (kg/m²)**			0.53
< 18.5	176 (7.3)	39 (7.6)	
18.5 – 25	1832 (75.5)	399 (77.3)	
≥ 25	416 (17.2)	78 (15.1)	
Missing	13	3	
**Parity**			0.99
0	1113 (45.7)	237 (45.7)	
1	908 (37.3)	193 (37.2)	
≥ 2	414 (17.0)	89 (17.1)	
Missing	2		
**History of miscarriage in a previous pregnancy**			0.50
No	2006 (82.4)	434 (83.6)	
Yes	429 (17.6)	85 (16.4)	

	Cohort with TTP not included in the biomonitoring (n=2437) n (%)	Subcohort with TTP (n=519) n (%)	p value
Missing	2		
**Oral contraceptive use before pregnancy attempt**			0.99
No	573 (23.6)	122 (23.5)	
Yes	1859 (76.4)	396 (76.5)	
Missing	5	1	
**Tobacco consumption at pregnancy attempt**			0.59
Non-smoker	1664 (69.6)	362 (70.8)	
Smoker	726 (30.4)	149 (29.2)	
Missing	47	8	
**Marital Status**			0.23
Lives with partners	2393 (98.3)	513 (99.0)	
Lives alone	41 (1.7)	5 (1.0)	
Missing	3	1	
**Medical assistance for fertility**			0.16
No	2217 (91.0)	482 (92.9)	
Yes	220 (9.0)	37 (7.1)	

Supplemental Material, Table S2. Relation between detection of metabolites detected in less than half of the sample

		EAA		EEAA		MPA		PrAA	
		**<LOD n (%)**	**≥LOD n (%)**	**<LOD n (%)**	**≥LOD n (%)**	**<LOD n (%)**	**≥LOD n (%)**	**<LOD n (%)**	**≥LOD n (%)**
**MAA**	**<LOD**	357 (71.7)	16 (72.2)	305 (71.4)	68 (73.9)	348 (71.0)	25 (86.2)	324 (70.7)	49 (80.3)
	**≥LOD**	141 (28.3)	5 (23.8)	122 (28.6)	24 (26.1)	142 (30.0)	4 (13.8)	134 (29.3)	12 (19.7)
		p[a]=0.65		p[a]=0.63		p[a]=0.08		p[a]=0.12	
**EAA**	**<LOD**			412 (96.5)	86 (93.5)	469 (95.7)	29 (100.0)	497 (96.1)	58 (95.1)
	**≥LOD**			15 (3.5)	6 (6.5)	21 (4.3)	0 (0.0)	18 (3.9)	3 (4.9)
				p[a]=0.09		p[a]=0.26		p[a]=0.71	
**EEAA**	**<LOD**					404 (82.4)	23 (79.3)	374 (81.7)	53 (86.9)
	**≥LOD**					86 (17.6)	6 (20.7)	84 (18.3)	8 (13.1)
						p[a]=0.67		p[a]=0.31	
**MPA**	**<LOD**							438 (95.6)	52 (85.2)
	**≥LOD**							20 (4.4)	9 (14.8)
								p[a]=0.0009	

[a] calculated using chi-square or Fisher’s exact

Supplemental Material, Table S3. Relation between ≥ LOD for metabolites detected in less than half the sample and concentration for metabolites detected in more than half the sample

		MEAA			BAA			PhAA		
		**GM[a] (mg/L)**	**Beta coef[b] Log MEAA**	**p**	**GM [a] (mg/L)**	**Beta coef[b] Log BAA**	**p**	**GM [a] (mg/L)**	**Beta coef[b] Log PhAA**	**p**
**MAA**	**<LOD**	0.10	Ref		0.11	Ref		0.36	Ref	
	**≥LOD**	0.18	0.39	0.002	0.12	-0.05	0.71	0.53	-0.06	0.63
**EAA**	**<LOD**	0.11	Ref		0.11	Ref		0.39	Ref	
	**≥LOD**	0.15	0.49	0.09	0.12	0.06	0.47	0.52	0.25	0.40
**EEAA**	**<LOD**	0.11	Ref		0.11	Ref		0.34	Ref	
	**≥LOD**	0.13	-0.14	0.36	0.12	0.04	0.65	0.82	0.36	0.02
**MPA**	**<LOD**	0.12	Ref		0.11	Ref		0.40	Ref	
	**≥LOD**	0.11	0.51	0.004	0.14	0.38	0.0001	0.41	-0.29	0.12
**PrAA**	**<LOD**	0.11	Ref		0.11	Ref		0.42	Ref	
	**≥LOD**	0.14	0.03	0.88	0.15	0.26	0.02	0.27	-0.04	0.88

[a] GM Geometric Mean

[b] calculated using Tobit regression

Supplemental Material, Table S4. Spearman rank correlations between urine concentration of MEAA, BAA and PhAA

	MEAA	BAA	PhAA
MEAA		r^2=-0.01 (p=0.84)	r^2=0.06 (p=0.19)
BAA	r^2=-0.01 (p=0.84)		r^2=-0.002 (p=0.96)
PhAA	r^2=0.06 (p=0.19)	r^2=-0.002 (p=0.96)	
